# Body Weight Measurement for Adjusting Diuretics Is Not Associated With Hospitalization and Mortality of Heart Failure Patients Under Home Medical Care

**DOI:** 10.7759/cureus.31800

**Published:** 2022-11-22

**Authors:** Hiroko Chuma, Yuki Kataoka

**Affiliations:** 1 Internal Medicine, Kyoto Min-Iren Asukai Hospital, Kyoto, JPN; 2 SRWS-PSG, Systematic Review Workshop Peer Support Group (SRWS-PSG), Osaka, JPN

**Keywords:** home medical care, hospitalization and mortality, diuretics, bodyweight measurement, heart failure

## Abstract

Introduction: The post-discharge readmission rate for heart failure (HF) is high. Although some previous studies have shown the efficacy of clinic-based patient education of regular weighing in preventing readmission; however, none of these studies were conducted in home care settings.

Methods: This retrospective observational study aimed to assess whether using body weight as an indicator for treating HF with diuretics reduced hospitalization and mortality in home medical care settings. We included 70 patients diagnosed with HF who were treated with diuretics in home medical care. Seventeen patients were in the body weight measurement group, and 53 were in the control group. The primary outcome was the time of the first hospitalization or death due to any cause. The secondary outcome was the time to first hospitalization or death due to HF.

Results: The crude hazard ratio (HR) for the primary outcome was 0.59 [95% confidence interval (CI), 0.33-1.1]. The crude HR for the secondary outcome was 0.95 (95% CI, 0.46-1.95). After adjusting for confounding factors, the results were found to be consistent.

Conclusion: Body weight measurement for treating HF was not associated with reduced hospitalization and mortality in medical care settings. Hence, physicians need other appropriate indicators.

## Introduction

The number of patients with heart failure (HF) has been increasing worldwide due to the aging population and an increase in lifestyle-related diseases. According to Savarese and Lund [1], the prevalence of HF has risen to over 1% in major countries, causing further increases in hospitalization rates and healthcare costs. Health expenditure for HF in the United States was around $31 billion in 2012 [1]. An epidemiological survey conducted in Sado City estimated that the number of HF patients in Japan will increase from 979,000 in 2005 to 1,284,000 in 2025 [2]. In a large-scale registry, JCARAE-CARD, in 2004, the mean age of the registered patients was 71 years [3], and in the most recent registry in Japan in 2014, JROADHF, the mean age was 78 years, and elderly patients over 75 years accounted for 68.9% of patients [4].

Most patients with HF are elderly and thus likely to receive home medical care. In a home care setting, resources are limited, and laboratory test results are not immediately available, though patients seem unstressed due to frequent interactions with physicians [5]. Therefore, adjusting the medication based on data that can be obtained quickly and easily at home is essential for HF treatment.

A large clinical trial in Japan showed that the readmission rate for HF one year post-discharge was as high as 25% [6]. Previous studies have shown that weight gain after discharge from HF treatment is associated with readmission [7-8]. Furthermore, some studies have reported that patient education, including weight records after hospital discharge, could reduce readmission or death due to any cause or HF [9-12].

However, under home medical care, no reports have shown that medical treatment based on body weight leads to reduced hospitalization and mortality in patients with HF. Therefore, this study assessed whether body weight could be used as a primary indicator for treating HF with diuretics to reduce hospitalization and mortality in home medical care settings.

## Materials and methods

Study design and setting

This retrospective observational study was conducted at the home visit center of Kyoto Min-Iren Askai Hospital. We followed the Strengthening Reporting of Observational Studies in Epidemiology (STROBE) guidelines in the reporting of this study.

Participants

The inclusion criteria for this study were as follows: (1) patients who underwent home visits by doctors from the above facility from April 2017 to March 2021; (2) patients aged 65 years and above; (3) patients who were clinically diagnosed with HF by previous doctors who referred patients to our facility and had clinical codes of HF, including I110, I130, I132, I500, I501, and I509, based on the ICD-10 classification at the first home visit; (4) patients treated with loop diuretics or mineralocorticoid receptor antagonists or an oral selective V2 receptor antagonist (tolvaptan); (5) patients who underwent activity of daily living (ADL) evaluation as per the criteria “the degree of independent living for disabled elderly” (Table 1) used for Long-Term Insurance System in Japan and whose ADL was B1 or better.

**Table 1:** The degree of independent living for disabled elderly: the criteria used to evaluate the degree for long-term care insurance system in Japan.

Rank	Explanation
J1	A person can go out by themself using the public transportation system
J2	A person can go out by themself in the neighborhood
A1	A person requires assistance to go out but can stay out of bed most of the day
A2	A person seldom goes out and has several rests in bed during the day
B1	A person needs some help in indoor life and spends most of the day in bed but can transfer to a wheelchair and get up for meals by themself
B2	A person needs some help in indoor life and spends most of the day in bed but can transfer to a wheelchair with assistance
C1	A person spends all day in bed but can turn over in bed without assistance
C2	A person spends all day in bed and cannot turn over without assistance

The exclusion criterion for this study was malignancy with a prognosis of less than one year. Due to the retrospective nature of this study, all available data were used. Patient-informed consent was obtained as an opt-out consent method on both the website and hospital notice board.

Main exposure

The patients were divided into the weight measurement group and the control group. Doctors did home visits basically twice a month in both groups. Those in the weight measurement group were weighed at least twice a month at each home visit; this was done at any time during the day. The type and amount of diuretics used were determined by the physicians.

Outcomes

The primary outcome was a composite of hospitalization or death due to any cause. The secondary outcome was the time to first hospitalization or death due to HF.

Ethical consideration

The study protocol was approved by the research ethics committee of the Kyoto Min-Iren Asukai Hospital.

Statistical analyses

Baseline characteristics were compared between the two groups using the Chi-square test or Fisher’s exact test for categorical variables. We used Welch’s t-test or the Wilcoxon rank-sum (exact) test for continuous variables. Categorical data were presented as frequencies and percentages. Normally distributed continuous variables were presented as means and standard deviation (SD), and those with non-normal distributions were presented as medians. Patients with missing data were excluded from the analysis. We followed up on patients' data from the date of the first home visit between April 2017 and March 2021 to the event date until the end of March 2022 or censored at the end of March 2022. Analyzed as the time to the first event, the Kaplan-Meier curve and log-rank test were used for the two-group comparison of the days until admission or death. Cox proportional hazard model was then used to estimate the confounding-adjusted hazard ratio (HR). All statistical analyses were performed using RStudio Cloud (R version 4.2.0).

## Results

**Figure 1:**
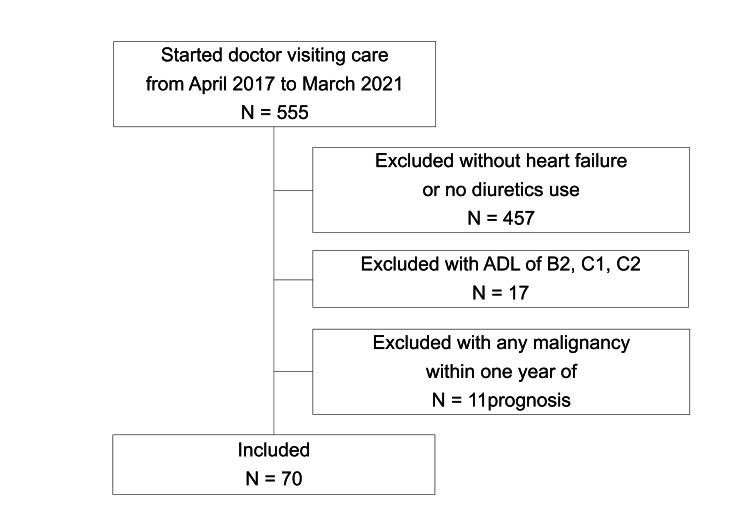
Participants and exclusion criteria. ADL, activities of daily living. B2 means a person needs some help in indoor life and spends most of the day in bed but can transfer to a wheelchair with assistance. C1 means a person spends all day in bed but can turn over in bed without assistance. C2 means a person spends all day in bed but cannot turn over in bed without assistance.

A patient flowchart is shown in Figure 1.

A total of 555 patients received home medical care between April 2017 and March 2021. Among them, we enrolled 70 patients with a mean age of 87 years (SD ± 7 years), of which 29 (41%) were men. Among the 70 patients, 17 were in the weight measurement group and 53 were in the control group. The baseline characteristics of the patients are provided in Table 2. In the control group, an additional 39% of patients had an ADL rank of B1 than in the weight measurement group. In addition, there were more NYHA class I patients but fewer NYHA class II and III patients in the control group than in the weight measurement group.

**Table 2:** Baseline characterlistics. *n (%); mean ± standard deviation; median (interquartile range) †Pearson's Chi-squared test; Welch two sample t-test; Fisher's exact test; Wilcoxon rank sum test; Wilcoxon rank sum exact test ‡The equation for Japanese is as follows; eGFR (mL/min/1.73 m^2^) = 194 × serum creatinine−1.094 × Age−0.287 × (0.739 if female) ADL, activities of daily living; NYHA, New York Heart Association; eGFR, estimated glomerular filtration rate; LVEF, left ventricular ejection fraction; BNP, brain natriuretic peptide; NT-proBNP, N-terminal prohormone of brain natriuretic peptide; MRA, mineralocorticoid receptor antagonist; AF, atrial fibrillation; IHD, ischemic heart disease

Characteristic	Overall, N = 70*	Control, N = 53*	Weight measurement, N = 17*	p-value^†^
Male	29 (41%)	23 (43%)	6 (35%)	0.6
Age (years)	87 ± 7	88 ± 6	85 ± 9	0.3
ADL				0.006
J2	6 (8.6%)	3 (5.7%)	3 (18%)	
A1	8 (11%)	7 (13%)	1 (5.9%)	
A2	27 (39%)	16 (30%)	11 (65%)	
B1	29 (41%)	27 (51%)	2 (12%)	
NYHA Classification				0.032
I	35 (50%)	31 (58%)	4 (24%)	
II	27 (39%)	17 (32%)	10 (59%)	
III	8 (11%)	5 (9.4%)	3 (18%)	
eGFR (ml/min/m^2^)^‡^	43 ± 17	44 ± 17	40±16	0.4
LVEF (%)	62 (51, 73)	62 (50, 73)	62 (59, 71)	0.8
(Missing)	3	2	1	
BNP (pg/mL)	143.3 (68.9, 269.8)	149.7 (67.8, 250.5)	143.3 (69.1, 294.1)	>0.9
(Missing)	11	11	0	
NT-proBNP (pg/mL)	1,168 (470, 3,420)	1,077 (440, 3,545)	1,793 (562, 3,058)	0.6
(Missing)	10	8	2	
Loop diuretics	66 (94%)	50 (94%)	16 (94%)	>0.9
MRA	30 (43%)	21 (40%)	9 (53%)	0.3
Tolvaptan	5 (7.1%)	2 (3.8%)	3 (18%)	0.088
AF	37 (53%)	25 (47%)	12(71%)	0.092
IHD	14 (20%)	9 (17%)	5(29%)	0.3

**Figure 2:**
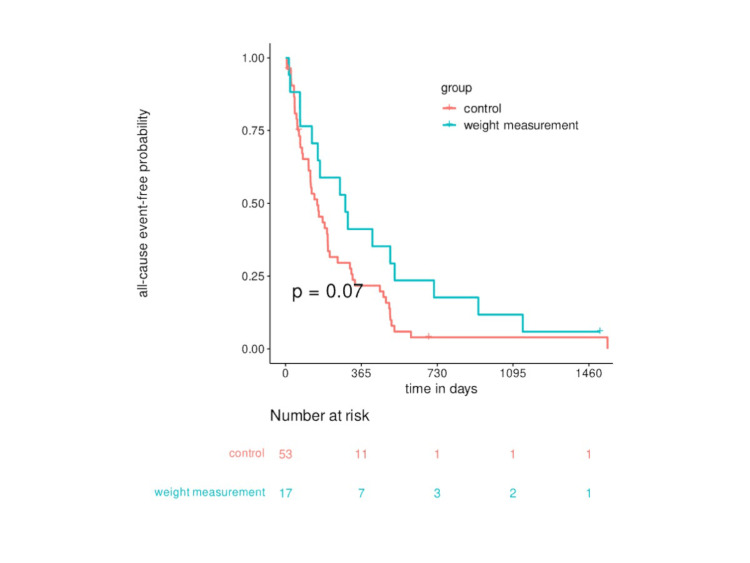
Kaplan-Meier curve for all-cause event-free survival.

Figure 2 shows the Kaplan-Meier curve for the probability of event-free survival due to all-cause hospitalization or death in the two groups. The median survival time of all patients was 288 days. At 365 days after the first home visit, the event-free probability of the control group was 22% and that of the weight measurement group was 41%. No statistically significant differences were observed between the two groups (p = 0.07). The HR was 0.59 (95% confidence interval [CI], 0.33-1.05), and the adjusted HR was 0.42 (95% CI, 0.19-0.93).

**Figure 3:**
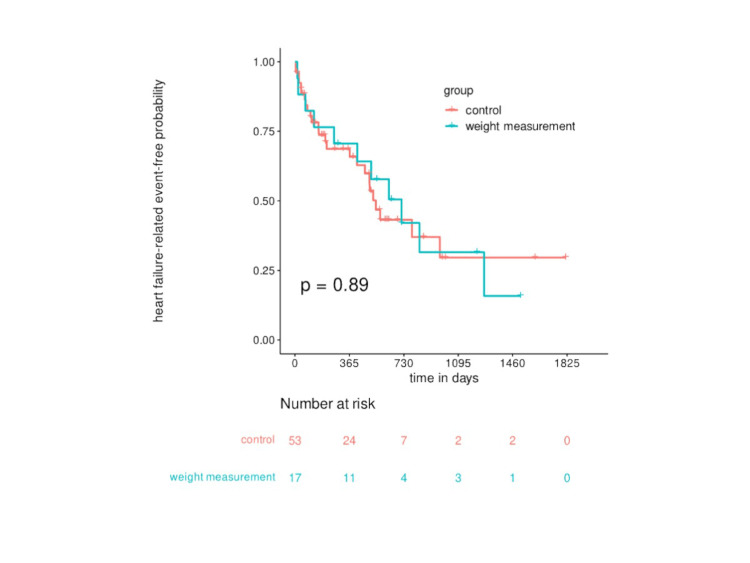
Kaplan-Meier curve for HF-related event-free survival. HF, heart failure

Figure 3 shows the Kaplan-Meier curve for the probability of event-free survival of hospitalization or death due to HF. The median survival time of all patients was 571 days. At 365 days after the first home visit, the event-free probability of the control group was 69% and that of the weight measurement group was 71%; no significant differences were observed between the two groups (p = 0.89). The HR was 0.95 (95% CI, 0.46-1.95), and the adjusted HR was 0.82 (95% CI, 0.31-2.14).

## Discussion

In this retrospective observational study, body weight measurements were not associated with reduced admission or death due to HF among patients with HF receiving home medical care. The results were the same for admission or death due to any cause.

Unlike in previous studies, adjusting diuretics based on body weight measurements was not associated with prognosis in patients with HF. There are three reasons for this discrepancy: the participant’s age, measurement intervals, and prescription frequencies.

The first reason is the difference in participants' ages. In this study, the patients were approximately 20 years older (mean age was in the 80s) than those in previous studies, where the outcome of patients with HF was improved by adjusting diuretics using body weight [9, 13]. In poor condition, older patients under home medical care often lose weight quickly as a reduction in their muscle and fat mass due to loss of appetite and reduced activity. Muscle wasting is commonly observed in patients with HF, particularly in older patients [14]. Loss of muscle and fat can offset weight gain due to fluid retention caused by HF. The result of a study showing that weight gain is not as sensitive as a screening method for HF deterioration supports this assumption [15]. We presume that the tendency to lose muscle and fat mass in older patients could be a possible reason for the unreliable body weight measurement.

Second, body weight was measured daily in previous studies. In an article that provided practical management recommendations for patients with HF based on the European Society of Cardiology guidelines, the patient guidance recommended self-monitoring, including daily weight measurements [16]. However, since most patients were older in our study, they had low ADL or cognitive decline and could not weigh themselves daily. Therefore, body weight was measured only on doctor visit days, essentially twice a month in many cases. The frequency of body weight measurements may have influenced the results.

Third, doctors in Japan can visit patients to change medications more frequently, if needed, than those in other countries. Some studies have reported that early physician follow-up after discharge reduces mortality and readmission rates [17-18]. Thus, the frequency of doctor visits may have influenced our results.

The results of our study suggest that attention should be paid not only to weight change but also to other findings when treating older patients with HF in home medical care settings. Although there is little evidence for improving prognosis, the 2021 ESC guidelines for HF advise self-monitoring symptoms for patients with HF, such as increasing dyspnea, edema, or sudden weight gain for self-management of diuretic dose [19]. In home medical care settings, dyspnea or edema may be indicators of HF treatment with diuretics, instead of body weight.

Our study has some limitations. First, we could not set a uniform clinical standard for HF for this study because previous doctors have diagnosed HF clinically. Second, this was a single-center retrospective cohort study, as such sample size of the weight measurement group was relatively small. Multi-center studies with larger sample sizes are needed to confirm results. Furthermore, prospective randomized controlled trials are required to assess whether the effect of weight measurement differs depending on the measurement frequency or the addition of other indicators.

## Conclusions

Physicians should not rely excessively on body weight when treating patients with homebound HF. Larger prospective studies are needed to identify appropriate indicators of diuretic therapy in older patients with HF under home medical care.
